# ﻿Five novel species of *Curvularia* (Pleosporales, Pleosporaceae) isolated from turfgrasses

**DOI:** 10.3897/mycokeys.125.168614

**Published:** 2025-11-21

**Authors:** Jia-Mei Zhao, Chuan-Xu Peng, Qiu-Yue Zhang, De-Wei Li, Lin Huang

**Affiliations:** 1 College of Forestry and Grassland, Nanjing Forestry University, Nanjing 210037, China Nanjing Forestry University Nanjing China; 2 Collaborative Innovation Center of Sustainable Forestry in Southern China, Nanjing 210037, China Collaborative Innovation Center of Sustainable Forestry in Southern China Nanjing China; 3 The Connecticut Agricultural Experiment Station Valley Laboratory, Windsor, CT 06095, USA The Connecticut Agricultural Experiment Station Valley Laboratory Windsor United States of America

**Keywords:** Fungal diversity, Helminthosporioid fungi, hyphomycetes, phylogeny, Pleosporaceae, taxonomy

## Abstract

*Curvularia*, a cosmopolitan fungal genus, occupies various ecological niches, but displays a pronounced tendency to colonise the leaves of plants. In this study, several fungal isolates with similar characteristics in the genus *Curvularia* were collected from leaf spots of turfgrasses (*Cynodon dactylon* and *Lolium perenne*) in Jiangsu Province, China. Based on the morphological characteristics and three locus phylogeny of the internal transcribed spacer (ITS) genes, glyceraldehyde-3-phosphate dehydrogenase (GAPDH) and translation elongation factor-1 alpha (*tef1*), five new species in *Curvularia*: *C. cynodontis*, *C. herbicola*, *C. loliicola*, *C. nanjingensis* and *C. xuanwuensis*, are described hereby. The present study contributes to the understanding of species diversity, taxonomy and phylogeny of *Curvularia* species in China.

## ﻿Introduction

Turfgrass, defined as artificially maintained vegetation, plays a crucial role in enhancing aesthetics, preventing soil erosion and supporting ecological balance in urban landscapes, sports and recreational settings (Braun et al. 2024). In China, the research on turfgrass breeding started relatively late, with only 1–2 varieties being registered annually and only about 20 of these varieties are considered suitable for lawn applications (Lai and Han 2022). As a result, cool-season turfgrasses, such as *Poa pratensis*, *Festuca arundinacea* and *Lolium perenne*, along with warm-season species like *Cynodon dactylon*, are predominantly imported (Asano and Ugaki 1994; Inokuma et al. 1998). Turfgrasses are susceptible to over 300 fungal pathogens that affect leaves, sheaths and roots. Notable diseases include brown patch, dollar spots, summer patch, powdery mildew, smut, *Pythium* blight, *Fusarium* blight, anthracnose and *Curvularia* leaf spots (Couch 1995; Toda et al. 2007; Kammerer et al. 2011).

The genus *Curvularia* Boedijn, belonging to Pleosporaceae, Pleosporales, Dothideomycetes, was established and typified by *C. lunata* (Wakker) Boedijn (Boedjin 1933). It is characterised by the intercalary and terminal conidiogenous cells and sympodial conidiophores. Most conidia of this genus are falcate or curved, though straight conidia also occur in some taxa (Manamgoda et al. 2015; Ferdinandez et al. 2023; van Vuuren et al. 2024; Ahmadpour et al. 2025). *Curvularia* species have a cosmopolitan distribution and inhabit diverse niches as plant pathogens, endophytes, saprobes or opportunistic human pathogens (Dransfield 1966; Almaguer et al. 2012; Manamgoda et al. 2015; Marin-Felix et al. 2017). Additionally, most of the species are reported from poaceous hosts (Smiley et al. 2005; Manamgoda et al. 2012; Tan et al. 2018; Marin et al. 2020; Tredway et al. 2023). It is a species-rich genus with 253 epithets and varieties in Index Fungorum (http://www.indexfungorum.org, accessed on 1 July 2025).

Morphologically, *Curvularia* was recognised as a genus often confused with *Bipolaris* Shoemaker (Sivanesan 1987; Tan et al. 2018; van Vuuren et al. 2024). These genera contain species with straight or curved conidia; the conidia of *Curvularia* show curvature due to disproportionately larger intermediate cells, whereas the curvature of *Bipolaris* is continuous along the entire length of the conidium. Though, generally, *Bipolaris* conidia are longer than *Curvularia* conidia, they are still difficult to distinguish (Manamgoda et al. 2014, 2015; Marin-Felix et al. 2020; Ahmadpour et al. 2025). On the other hand, the sexual morphs of these genera are similar and previously classified as *Cochliobolus* Drechsler (synonymous with *Pseudocochliobolus* Tsuda, Ueyama & Nishih.), characterised by brown or black, globose ascomata (pseudothecia), bitunicate and cylindrical asci and filiform or flagelliform, hyaline ascospores, which are loosely organised into a helix or parallel in the ascus (Sivanesan 1987; Manamgoda et al. 2014, 2015). However, it is rare to find sexual morphs in nature (Sivanesan 1987).

Due to the difficulty in distinguishing these genera using morphological characters, species recognition in these genera relies on molecular analyses (Manamgoda et al. 2014). Although the internal transcribed spacer (ITS) region has been selected as the primary fungal barcode marker, it has low resolution for *Curvularia*. Therefore, for speciation of *Curvularia*, multi-locus sequence analyses of ITS and the partial gene regions of glyceraldehyde-3-phosphate dehydrogenase (GAPDH) and translation elongation factor 1 alpha (*tef1*) genes have been proposed (Manamgoda et al. 2012; Marin-Felix et al. 2017, 2020; Tan et al. 2018). Numerous studies have shown that *Curvularia* is currently recognised as a well-defined monophyletic genus (Manamgoda et al. 2015; Marin-Felix et al. 2020).

During the extensive investigations conducted to collect turfgrass pathogens in Nanjing, China, several *Curvularia* taxa exhibiting typical characteristics were successfully isolated. The primary objective of this study was to determine the identities of these newly-collected *Curvularia* species through morphological studies and phylogenetic analyses, while also elucidating their phylogenetic relationships.

## ﻿Materials and methods

### ﻿Sample collection and potential fungal pathogen isolation

Samples were collected from leaf blight of grassland in six parks across main urban districts of Nanjing, Jiangsu Province, China. Within each park, seven to ten 1 × 1 m quadrats were surveyed. The comprehensive data of each sampling site are listed in Suppl. material 1: table S1, including specific location, survey date, geographic coordinates (latitude, longitude), turf area and turfgrass variety.

Small sections (2 × 3 mm) were cut from the margins of infected tissues and surface sterilised in 75% alcohol for 30 s, then in 1% sodium hypochlorite (NaOCl) for 90 s, followed by three rinses with sterile water (Huang et al. 2016), then blotted dry with sterilised filter paper, placed on Petri plates with 2% potato dextrose agar (PDA) and 100 mg/l ampicillin and then cultured for 3 days at 25 °C in the dark. Fungal isolates were purified with the monosporic isolation method described by using the spores produced with liquid cultures (Li et al. 2007). Single-spore isolates were maintained on PDA plates. The obtained isolates were stored in the Forest Pathology Laboratory at Nanjing Forestry University. Holotype specimens of new species from this study were deposited at the China Forestry Culture Collection Center (CFCC, https://cfcc.caf.ac.cn/), Chinese Academy of Forestry, Beijing, China.

### ﻿Morphological analyses

The morphology of the new species identified in this study was analysed, based on fruiting bodies naturally formed on leaves and PDA plates. Micromorphological structures were photographed using the Zeiss stereomicroscope. The shape, colour and size of conidiophores and conidia were observed using a ZEISS Axio Scope 5 microscope (ZEISS, Germany) with differential interference contrast (DIC) optics. For each structure, 30 measurements were made. Colours were determined using Kornerup and Wanscher (1978). Photo plates were made by Adobe Photoshop 2021. For spore measurements, 30 spores were randomly selected. The results are presented as maximum and minimum values (in parentheses), along with the range expressed as the (min.–) X–SD–X + SD (–max.).

### ﻿Phylogenetic analyses

Genomic DNA of 26 isolates was extracted using a modified CTAB method (Damm et al. 2008). The fungal plugs of each isolate were grown on the PDA plates for 5 days and then collected in a 2 ml tube. Then, 500 µl of chloroform and 500 µl of hexadecyltrimethyl ammonium bromide (CTAB) extraction buffer (0.2 M Tris, 1.4 M NaCl, 20 mM EDTA, 0.2 g/l CTAB) were added into the tubes, which were placed in a shaker at 30 °C at 200 rpm for 1.5 h. The mixture was centrifuged at 15,800 × g for 10 min. Then, 300 µl of the supernatant was transferred into a new tube and 600 µl of 100% ethanol were added. The suspension was centrifuged at 15,800 × g for 5 min. At that point, 600 µl of 75% ethanol were added into the precipitate. The suspension was centrifuged at 15,800 × g for 5 min and the supernatant was discarded. The DNA pellet was dried and re-suspended in 30 µl ddH_2_O. The quality of the extracted DNA was assessed using a microvolume spectrophotometer.

To amplify the ITS, GAPDH and *tef1* loci, the following primer pairs were used: ITS1/ITS4 (White et al. 1990), gpd1/gpd2 (Berbee et al. 1999) and *tef1*-983F/*tef1*-2218R (Rehner and Buckley 2005), respectively. The primer sequences are listed in Suppl. material 1: table S2. The parameters for PCR protocol were as follows: 94 °C for 4 min, followed by 35 cycles of 30 s at 94 °C, 45 s at 55–60 °C and 45 s at 72 °C, which were used in a touch down PCR (Korbie and Mattick 2008). A final extension step was conducted for 10 min at 72 °C. The primers were synthesised by Sangon Biotech, Nanjing, Jiangsu Province, China. The amplified products were sequenced in Shanghai Jie Li Biotechnology Co., Ltd.

To include a more comprehensive range of species, we chose specific genes/regions for our study, based on research by Ahmadpour et al. (2025). These were as follows: ITS + GAPDH + *tef1* for *Curvularia* (Table 1). The dataset was then aligned separately using MAFFT v.74 (http://mafft.cbrc.jp/alignment/server/) (Katoh et al. 2019) with the G-INS-I iterative refinement algorithm and optimised manually in BioEdit v.7.0.5.3 (Hall 1999). The separate alignments were then concatenated using PhyloSuite v.1.2.2 (Zhang et al. 2020a).

**Table 1.** Cultures, specimens and NCBI accession numbers included in this study. ^T^ indicates ex-type strains.

Species	Isolate/Culture collection	Host/Substratum	Country	GenBank accessions		
				ITS	GAPDH	*tef1*
*Bipolaris maydis*	CBS 137271 ^T^	*Zea mays*	USA	AF071325	KM034846	KM093794
*Curvularia aeria*	CBS 294.61 ^T^	Air	Brazil	HF934910	HG779148	–
*C. affinis*	CBS 154.34 ^T^	Unknown	Indonesia	KJ909780	KM230401	KM196566
*C. akaii*	CBS 318.86 ^T^	Unknown	Japan	LT631340	LT715797	–
*C. akaiiensis*	BRIP 16080 ^T^	Unknown	India	KJ415539	KJ415407	KJ415453
*C. alcornii*	MFLUCC 10-0703 ^T^	*Zea mays*	Thailand	JX256420	JX276433	JX266589
*C. algeriensis*	CBS 150506 ^T^	*Zea mays*	Algeria	OP218257	OP223173	OP223186
*C. americana*	UTHSC 08-3414 ^T^	Human ankle	USA	HE861833	HF565488	–
*C. andropogonis*	CBS 186.49 ^T^	*Andropogon nardus*	Indonesia	LT631354	LT715835	–
*C. angsiewkeeae*	BRIP 72449a^T^	*Scleria* sp.	Australia	OK638993	OK655929	OK655924
*C. annelliconidiophori*	CGMCC 3.19352 ^T^	*Saccharum officinarum*	China	MN215641	MN264077	MN263935
*C. asiatica*	MFLUCC 10-0711 ^T^	*Panicum* sp.	Thailand	JX256424	JX276436	JX266593
*C. aurantia*	USJCC-0096 ^T^	*Zea mays*	Sri Lanka	OQ275217	OQ269628	OQ332409
*C. australiensis*	BRIP 12044 ^T^	*Oryza sativa*	Australia	KJ415540	KJ415406	KJ415452
*C. australis*	BRIP 12521 ^T^	*Sporobolus caroli*	Australia	KJ415541	KJ415405	KJ415451
*C. austriaca*	CBS 102694 ^T^	Human nasal cavity	Austria	MN688802	MN688829	MN688856
*C. bannonii*	BRIP 16732 ^T^	*Jacquemontia tamnifolia*	USA	KJ415542	KJ415404	KJ415450
*C. beasleyi*	BRIP 10972 ^T^	*Chloris gayana*	Australia	MH414892	MH433638	MH433654
*C. beasleyi*	BRIP 15854	*Leersia hexandra*	Australia	MH414893	MH433639	MH433655
*C. beerburrumensis*	BRIP 12942 ^T^	*Eragrostis bahiensis*	Australia	MH414895	MH433634	MH433657
*C. boeremae*	IMI 164633 ^T^	*Portulaca oleracea*	India	MH414911	MH433641	–
*C. borreriae*	CBS 859.73 ^T^	Volcanic ash soil	Chile	LT631355	LT715838	–
*C. bothriochloae*	BRIP 12522 ^T^	*Bothriochloa bladhii*	Australia	KJ415543	KJ415403	KJ415449
*C. boudouaouensis*	CBS 150515 ^T^	*Zea mays*	Algeria	OP218258	OP223175	OP223187
*C. brachyspora*	CBS 186.50 ^T^	Soil	Java	HG778983	KM061784	KM230405
*C. buchloes*	CBS 246.49 ^T^	*Buchloe dactyloides*	USA	KJ909765	KM061789	KM196588
*C. cactivora*	CBS 580.74	*Member of Cactaceae*	Republic of Suriname	MN688803	MN688830	MN688857
*C. canadensis*	CBS 109239 ^T^	*Overwintered grass*	Canada	MN688804	MN688831	MN688858
*C. caricae-papayae*	CBS 135941 ^T^	*Carica papaya*	India	LT631350	LT715816	–
*C. caspica*	IRAN 4275C^T^	*Eleocharis* sp.	Iran	PP593896	PP661368	PP661351
*C. caspica*	FCCUU 1103	*Cyperus* sp.	Iran	PP593897	PP661369	PP661352
*C. caspica*	FCCUU 1104	*Fimbristylis* sp.	Iran	PP593898	PP661370	PP661353
*C. chiangmaiensis*	CPC 28829 ^T^	*Zea mays*	Thailand	MF490814	MF490836	MF490857
*C. chiangraiensis*	MFLUCC 22-0091 ^T^	Soil	Thailand	OP581428	OP859013	OP859017
*C. chlamydospora*	UTHSC 07-2764 ^T^	Human toenail	USA	HG779021	HG779151	–
*C. chonburiensis*	MFLUCC 16-0375 ^T^	*Pandanus* sp.	Thailand	MH275055	MH412747	–
*C. chuasooengiae*	BRIP 72482a ^T^	*Scleria* sp.	Australia	OK638997	OK655933	–
*C. clavata*	BRIP 61680b	*Oryza rufipogon*	Australia	KU552205	KU552167	KU552159
*C. coatesiae*	BRIP 24261 ^T^	*Litchi chinensis*	Australia	MH414897	MH433636	MH433659
*C. coicicola*	HSAUP 990901	*Coix lacryma-jobi*	China	AB453880	–	–
*C. coicis*	CBS 192.29 ^T^	*Coix lacryma-jobi*	Japan	HF934917	HG779130	JN601006
*C. coimbatorensis*	SZMC 22225 ^T^	Human cornea	India	MN628310	MN628306	MN628302
*C. colbranii*	BRIP 13066 ^T^	*Crinum zeylanicum*	Australia	MH414898	MH433642	MH433660
*C. comoriensis*	CBS 110673	Unknown	Unknown	LT631357	LT715841	–
*C. crassiseptata*	CBS 503.90 ^T^	Plant material	Nigeria	LT631310	LT715882	MN688859
*C. crustacea*	BRIP 13524 ^T^	*Sporobolus* sp.	Indonesia	KJ415544	KJ415402	KJ415448
*C. curculiginis*	YZU 181230	*Curculigo capitulata*	China	MK507796	MK507794	MK507795
*C. cymbopogonis*	CBS 419.78 ^T^	*Yucca* sp.	Netherlands	HG778985	HG779129	–
***C. cynodontis***	**SCM16-22^T^**	**Leaves of *Cynodon dactylon***	**China**	**PV973428**	**PV995188**	**PV995204**
***C. cynodontis***	**YZ26-27**	**Leaves of *Cynodon dactylon***	**China**	**PV973429**	**PV995189**	**PV995206**
***C. cynodontis***	**XW6-23**	**Leaves of *Cynodon dactylon***	**China**	**PV973430**	**PV995190**	**PV995205**
*C. cyperi*	IRAN 4274C ^T^	*Cyperus* sp.	Iran	PP593899	PP661371	PP661354
*C. cyperi*	FCCUU 1101	*Fimbristylis* sp.	Iran	PP593900	PP661372	PP661355
*C. cyperi*	FCCUU 1102	*Isolepis* sp.	Iran	PP593901	PP661373	PP661356
*C. dactyloctenicola*	CPC 28810 ^T^	*Dactyloctenium aegyptium*	Thailand	MF490815	MF490837	MF490858
*C. dactyloctenii*	BRIP 12846 ^T^	*Dactyloctenium radulans*	Australia	KJ415545	KJ415401	KJ415447
*C. dactyloctenii*	7938-9	*Eureka blueberry*	China	AF158106	AF081376	–
*C. deightonii*	CBS 537.70 ^T^	*Sorghum vulgare*	Denmark	LT631356	LT715839	–
*C. deserticola*	CBS 151410 ^T^	*Stipagrostis ciliata*	Namibia	ON074985	ON355399	ON355360
*C. determinata*	CGMCC 3.19340 ^T^	*Saccharum officinarum*	China	MN215653	MN264088	MN263947
*C. eleusinicola*	USJCC-0005 ^T^	*Eleusine coracana*	Sri Lanka	MT262877	MT393583	MT432925
*C. elliptiformis*	CGMCC 3.19351 ^T^	*Saccharum officinarum*	China	MN215656	MN264091	MN263950
*C. ellisii*	CBS 193.62 ^T^	Air	Pakistan	JN192375	JN600963	JN601007
*C. eragrostidicola*	BRIP 12538 ^T^	*Eragrostis pilosa*	Australia	MH414899	MH433643	MH433661
*C. eragrostidis*	CBS 189.48 ^T^	*Sorghum* sp.	Java	HG778986	HG779154	–
*C. falsilunata*	CGMCC 3.19329 ^T^	*Saccharum officinarum*	China	MN215660	MN264093	MN263954
*C. flexuosa*	CGMCC 3.19447 ^T^	*Saccharum officinarum*	China	MN215663	MN264096	MN263957
*C. frankliniae*	BRIP 72476a^T^	*Sorghum timorense*	Australia	OK638995	OK655931	OK655926
*C. fraserae*	BRIP 64708a^T^	*Bothriochloa insculpta*	Australia	OM809867	OM721558	OM714552
*C. geniculata*	CBS 187.50 ^T^	*Andropogon sorghum*	Indonesia	KJ909781	KM083609	KM230410
*C. gladioli*	CBS 210.79	*Gladiolus* sp.	Romania	LT631345	LT715802	–
*C. gobabebensis*	CBS 149140 ^T^	*Stipagrostis ciliata*	Namibia	ON074797	ON355381	ON355347
*C. gobabebensis*	CMW-IA 6921	*Stipagrostis ciliata*	Namibia	ON332848	ON355373	ON355344
*C. graminicola*	BRIP 23186a^T^	*Aristida ingrata*	Australia	JN192376	JN600964	JN601008
***C. herbicola***	**JHS14-23**	**Leaves of *Lolium perenne***	**China**	**PV973431**	**PV995191**	**PV995207**
***C. herbicola***	**YYH23-17**	**Leaves of *Cynodon dactylon***	**China**	**PV973432**	**PV995192**	**PV995208**
***C. herbicola***	**XW1-15^T^**	**Leaves of *Cynodon dactylon***	**China**	**PV973433**	**PV995193**	**PV995209**
*C. guangxiensis*	CGMCC 3.19330 ^T^	*Saccharum officinarum*	China	MN215667	MN264100	MN263961
*C. gudauskasii*	DAOM 165085	Unknown	Unknown	AF071338	AF081393	–
*C. harveyi*	BRIP 57412 ^T^	*Triticum aestivum*	Australia	KJ415546	KJ415400	KJ415446
*C. hawaiiensis*	BRIP 11987 ^T^	*Oryza sativa*	USA	KJ415547	KJ415399	KJ415445
*C. heteropogonicola*	BRIP 14579 ^T^	*Heteropogon contortus*	India	KJ415548	KJ415398	KJ415444
*C. heteropogonis*	CBS 284.91 ^T^	*Heteropogon contortus*	Australia	KJ415549	JN600969	JN601013
*C. hominis*	UTHSC 09-464 ^T^	Human cornea	USA	HG779011	HG779106	–
*C. homomorpha*	CBS 156.60 ^T^	Air	USA	JN192380	JN600970	JN601014
*C. huamulaniae*	BRIP 10936a ^T^	Air	Australia	OR130931	OR135531	OR135532
*C. hustoniae*	BRIP 72486a ^T^	*Heteropogon triticeus*	Australia	OK638999	OK655935	OK655928
*C. inaequalis*	CBS 102.42 T	Soil	France	KJ922375	KM061787	KM196574
*C. intermedia*	CBS 334.64	*Avena versicolor*	USA	HG778991	HG779155	–
*C. intermedia*	XY-3	*Eureka blueberry*	China	OQ300329	OQ338159	OQ338160
*C. iranica*	IRAN 3487C ^T^	*Bougainvillea spectabilis*	Iran	MT551122	MN266487	MN266490
*C. ischaemi*	CBS 630.82 ^T^	*Ischaemum indicum*	Solomon Islands	HG778992	HG779131	–
*C. joliotcurieae*	BRIP 14448a ^T^	*Triticum aestivum*	Australia	OQ917073	OQ889556	OQ889557
*C. kenpeggii*	BRIP 14530 ^T^	*Triticum aestivum*	Australia	MH414900	MH433644	MH433662
*C. khuzestanica*	CBS 144736 ^T^	*Atriplex lentiformis*	Iran	MH688044	MH688043	–
*C. khuzestanica*	SCUA-11C-2	*Atriplex lentiformis*	Iran	MH688046	MH688045	–
*C. kusanoi*	CBS 137.29 ^T^	*Eragrostis major*	Japan	JN192381	LT715862	KM196592
*C. lamingtonensis*	BRIP 12259 ^T^	*Microlaena stipoides*	Australia	MH414901	MH433645	MH433663
***C. loliicola***	**XW9-5-1^T^**	**Leaves of *Lolium perenne***	**China**	**PV973443**	**PV995201**	**PV995217**
***C. loliicola***	**XW9-5-2**	**Leaves of *Lolium perenne***	**China**	**PV973441**	**PV995202**	**PV995218**
***C. loliicola***	**XW9-5-3**	**Leaves of *Lolium perenne***	**China**	**PV973442**	**PV995203**	**PV995219**
*C. lolii*	CMAA 1785 ^T^	*Lolium multiflorum*	Brazil	MT849336	MT889299	MT881706
*C. lonarensis*	CBS 140569 ^T^	*Lonar lake*	India	KT315408	KY007019	–
*C. lunata*	CBS 730.96 ^T^	Human lung biopsy	USA	JX256429	JX276441	JX266596
*C. lycopersici*	Strain 11	*Solanum lycopersicum*	Egypt	KY883347	KY883345	–
*C. malina*	CBS 131274 ^T^	*Zoysia matrella*	USA	JF812154	KP153179	KR493095
*C. manamgodae*	CGMCC 3.19446 ^T^	*Saccharum officinarum*	China	MN215677	MN264110	MN263971
*C. maraisii*	CBS 149143 ^T^	Soil	Namibia	OR471647	ON355439	OR486044
*C. mebaldsii*	BRIP 12900 ^T^	*Cynodon transvaalensis*	Australia	MH414902	MH433647	MH433664
*C. mebaldsii*	CMW-IA 6956	*Stipagrostis ciliata*	Namibia	ON644443	ON661549	–
*C. micrairae*	BRIP 17068a^T^	*Micraira subulifolia*	Australia	OM421618	OM373204	OM373205
*C. micropus*	CBS 127235 ^T^	*Paspalum notatum*	USA	HE792934	LT715859	–
*C. microspora*	GUCC 6272 ^T^	*Hippeastrum striatum*	China	MF139088	MF139106	MF139115
*C. millisiae*	BRIP 71718a ^T^	*Cyperus aromaticus*	Australia	OK661031	OK636415	OK636413
*C. miyakei*	CBS 197.29 ^T^	*Eragrostis pilosa*	Japan	KJ909770	KM083611	KM196568
*C. moringae*	CPC 38873 ^T^	*Moringa ovalifolia*	Namibia	MW175363	MW173105	–
*C. mosaddeghii*	IRAN 3131C^T^	*Syzygium cumini*	Iran	MG846737	MH392155	MH392152
*C. muehlenbeckiae*	CBS 144.63 ^T^	*Muehlenbeckia* sp.	India	HG779002	HG779108	–
*C. namibensis*	CBS 149144 ^T^	*Stipagrostis ciliata*	Namibia	ON074819	ON355384	ON355350
***C. nanjingensis***	**YZ25-10-2-1^T^**	**Leaves of *Cynodon dactylon***	**China**	**PV973438**	**PV995198**	**PV995214**
***C. nanjingensis***	**YZ25-10-2-2**	**Leaves of *Cynodon dactylon***	**China**	**PV973439**	**PV995199**	**PV995215**
***C. nanjingensis***	**YZ25-10-2-3**	**Leaves of *Cynodon dactylon***	**China**	**PV973440**	**PV995200**	**PV995216**
*C. nanningensis*	GUCC 11005 ^T^	*Cymbopogon citratus*	China	MH885321	MH980005	MH980011
*C. neergaardii*	BRIP 12919 ^T^	*Oryza sativa*	Ghana	KJ415550	KJ415397	KJ415443
*C. neoindica*	IMI 129790 ^T^	*Brassica nigra*	India	MH414910	MH433649	MH433667
*C. nicotiae*	BRIP 11983 ^T^	Soil	Algeria	KJ415551	KJ415396	KJ415442
*C. nodosa*	CPC 28800 ^T^	Digitaria ciliaris	Thailand	MF490816	MF490838	MF490859
*C. nuciferae*	YzU 231509	Nelumbo nucifera	China	OR888819	PP066825	PP792703
*C. nuciferae*	YzU 231510	Nelumbo nucifera	China	OR888818	PP066826	PP792704
*C. nodosa*	CPC 28801	–	Thailand	MF490817	MF490839	MF490860
*C. nodulosa*	CBS 160.58	*Eleusine indica*	USA	JN601033	JN600975	JN601019
*C. nodulosa*	IRAN 4804C	*Eleusine indica*	Iran	PP593892	PP661366	–
*C. nuciferae*	YzU 231509	*Nelumbo nucifera*	China	OR888819	PP066825	PP792703
*C. nuciferae*	YzU 231510	*Nelumbo nucifera*	China	OR888818	PP066826	PP792704
*C. oryzae*	CBS 169.53 ^T^	*Oryza sativa*	Vietnam	KP400650	HG779156	KM196590
*C. oryzae*	IRAN 5044C	Unknown	Iran	PP593893	PP661367	–
*C. oryzae-sativae*	CBS 127725 ^T^	*Oryza sativa*	Argentina	MN688808	MN688835	MN688863
*C. ovariicola*	CBS 470.90 ^T^	*Eragrostis interrupta*	Australia	MN688809	MN688836	–
*C. pallescens*	CBS 156.35 ^T^	Air	Indonesia	KJ922380	KM083606	KM196570
*C. palmicola*	MFLUCC 14-0404 ^T^	*Acoelorrhaphe wrightii*	Thailand	MF621582	–	–
*C. pandanicola*	MFLUCC 15-0746 ^T^	*Pandanus* sp.	Thailand	MH275056	MH412748	MH412763
*C. panici-maximi*	USJCC-0006 ^T^	*Panicum maximum*	Sri Lanka	MN044757	MN053040	MN053009
*C. papendorfii*	CBS 308.67 ^T^	*Acacia karroo*	South Africa	KJ909774	KM083617	KM196594
*C. paraverruculosa*	FMR 17656 ^T^	Soil	Mexico	LR736641	LR736646	LR736649
*C. patereae*	CBS 198.87 ^T^	*Triticum durum*	Argentina	MN688810	MN688837	MN688864
*C. penniseti*	CBS 528.70	Unknown	Unknown	MH859833	LT715840	–
*C. perotidis*	CBS 350.90 ^T^	*Perotis rara*	Australia	HG778995	HG779138	KM230407
*C. petersonii*	BRIP 14642 ^T^	*Dactyloctenium aegyptium*	Australia	MH414905	MH433650	MH433668
*C. phaeospara*	CGMCC 3.19448 ^T^	*Saccharum officinarum*	China	MN215686	MN264118	MN263980
*C. pisi*	CBS 190.48 ^T^	*Pisum sativum*	Canada	KY905678	KY905690	KY905697
*C. plantarum*	CGMCC 3.19342 ^T^	*Saccharum officinarum*	China	MN215688	MN264120	MN263982
*C. platzii*	BRIP 27703b ^T^	*Cenchrus clandestinum*	Australia	MH414906	MH433651	MH433669
*C. polytrata*	CGMCC 3.19338 ^T^	*Saccharum officinarum*	China	MN215691	MN264123	MN263984
*C. prasadii*	CBS 143.64 ^T^	*Jasminum sambac*	India	KJ922373	KM061785	KM230408
*C. protuberans*	CGMCC 3.19360 ^T^	*Saccharum officinarum*	China	MN215693	MN264125	MN263986
*C. protuberata*	CBS 376.65 ^T^	*Deschampsia flexuosa*	Scotland	KJ922376	KM083605	KM196576
*C. pseudobrachyspora*	CPC 28808 ^T^	*Eleusine indica*	Thailand	MF490819	MF490841	MF490862
*C. pseudoclavata*	CBS 539.70 ^T^	*Oryza sativa*	Denmark	MN688817	MN688844	MN688869
*C. pseudoellisii*	CBS 298.80 ^T^	*Sorghum bicolor*	Sudan	MN688818	MN688845	MN688870
*C. pseudointermedia*	CBS 553.89 ^T^	*Cultivated pasture soil*	Brazil	MN688819	MN688846	MN688871
*C. pseudolunata*	UTHSC 09-2092 ^T^	Human nasal sinus	USA	HE861842	HF565459	–
*C. pseudoprotuberata*	CBS 385.69 ^T^	Soil under Thuja occidentalis	Canada	MN688821	MN688848	MN688873
*C. pseudorobusta*	UTHSC 08-3458	Human nasal sinus	USA	HE861838	HF565476	–
*C. radicicola*	CGMCC 3.19327 ^T^	*Saccharum officinarum*	China	MN215699	MN264131	MN263992
*C. radici-foliigena*	CGMCC 3.19328 ^T^	*Saccharum officinarum*	China	MN215695	MN264127	MN263988
*C. ravenelii*	BRIP 13165 ^T^	*Sporobolus fertilis*	Australia	JN192386	JN600978	JN601024
*C. reesii*	BRIP 4358 ^T^	Air	Australia	MH414907	MH433637	MH433670
*C. richardiae*	BRIP 4371 ^T^	*Richardia brasiliensis*	Australia	KJ415555	KJ415391	KJ415438
*C. robusta*	CBS 624.68 ^T^	*Dichanthium annulatum*	USA	KJ909783	KM083613	KM196577
*C. rouhanii*	CBS 144674 ^T^	*Syngonium vellozianum*	Iran	KX139030	MG428694	MG428687
*C. rouhanii*	CN022H5	*Stipagrostis ciliata*	Namibia	ON074966	ON355388	ON355353
*C. ryleyi*	BRIP 12554 ^T^	*Sporobolus creber*	Australia	KJ415556	KJ415390	KJ415437
*C. saccharicola*	CGMCC 3.19344 ^T^	*Saccharum officinarum*	China	MN215701	MN264133	MN263994
*C. sacchari-officinarum*	CGMCC 3.19331 ^T^	*Saccharum officinarum*	China	MN215705	MN264137	MN263998
*C. senegalensis*	CBS 149.71	Unknown	Nigeria	HG779001	HG779128	–
*C. sesuvii*	CGMCC 3.9578 ^T^	*Sesuvium portulacastrum*	China	EF175940	–	–
*C. shahidchamranensis*	IRAN 3133C^T^	Soil	Iran	MH550084	MH550083	–
*C. sichuanensis*	BN9	Air	China	MH483998	–	–
*C. siddiquii*	CBS 196.62 ^T^	Air	Pakistan	MN688823	MN688850	–
*C. simmonsii*	USJCC-0002 ^T^	*Panicum maximum*	Sri Lanka	MN044753	MN053011	MN053005
*C. soli*	CBS 222.96 ^T^	Soil	Papua New Guinea	KY905679	KY905691	KY905698
*C. sorghina*	BRIP 15900 ^T^	*Sorghum bicolor*	Australia	KJ415558	KJ415388	KJ415435
*C. spicifera*	CBS 274.52	Soil	Spain	JN192387	JN600979	JN601023
*C. spicifera*	AT-102	*corn*	Northern Algeria	OP218259	OP223176	OP223188
*C. sporobolicola*	BRIP 23040b ^T^	*Sporobolus australasicus*	Australia	MH414908	MH433652	MH433671
*C. stipagrostidicola*	CBS 149150 ^T^	*Stipagrostis ciliata*	Namibia	ON332838	ON355415	ON355368
*C. stenotaphri*	BRIP 71303 ^T^	*Stenotaphrum secundatum*	Australia	MZ681952	MZ695824	MZ695819
*C. subpapendorfii*	CBS 656.74 ^T^	Soil	Egypt	KJ909777	KM061791	KM196585
*C. suttoniae*	FMR 10992 ^T^	Human leg wound	USA	HE861828	HF565479	LR736651
*C. tabaci*	YzU 221481	Nicotiana tabacum	China	OR888817	PP066824	OR818398
*C. tabaci*	YzU 221482	Nicotiana tabacum	China	PP601257	PP779503	PP779504
*C. tabaci*	YzU 221481	Nicotiana tabacum	China	OR888817	PP066824	OR818398
*C. tabaci*	YzU 221482	Nicotiana tabacum	China	PP601257	PP779503	PP779504
*C. tamilnaduensis*	SZMC 22226 ^T^	Human cornea	India	MN628311	MN628307	MN628303
*C. tanzanica*	IMI 507176 ^T^	*Cyperus aromaticus*	Tanzania	MW396857	MW388669	–
*C. templetoniae*	BRIP 72453a ^T^	*Hyparrhenia hirta*	Australia	OK638994	OK655930	OK655925
*C. thailandicum*	MFLUCC 15-0747 ^T^	*Pandanus* sp.	Thailand	MH275057	MH412749	MH412764
*C. tribuli*	CBS 126975 ^T^	*Tribulus terrestris*	South Africa	MN688825	MN688852	MN688875
*C. trifolii*	ICMP 6149	*Setaria glauca*	New Zealand	KM230395	KM083607	JX266600
*C. tripogonis*	BRIP 12375 ^T^	*Tripogon loliiformis*	Australia	JN192388	JN600980	JN601025
*C. tropicalis*	BRIP 14834 ^T^	*Coffea arabica*	India	KJ415559	KJ415387	KJ415434
*C. tsudae*	ATCC 44764 ^T^	*Chloris gayana*	Japan	KC424596	KC747745	KC503940
*C. tuberculata*	CBS 146.63 ^T^	*Zea mays*	India	JX256433	JX276445	JX266599
*C. umbiliciformis*	CGMCC 3.19346 ^T^	*Saccharum officinarum*	China	MN215711	MN264142	MN264004
*C. uncinata*	CBS 221.52 ^T^	*Oryza sativa*	Vietnam	HG779024	HG779134	–
*C. variabilis*	CPC 28815 ^T^	*Chloris barbata*	Thailand	MF490822	MF490844	MF490865
*C. verruciformis*	CBS 537.75	*Vanellus miles*	NewZealand	HG779026	HG779133	–
*C. verrucosa*	CBS 422.93	Air	Cuba	MN688826	MN688853	MN688876
*C. verruculosa*	CBS 150.63	*Punica granatum*	India	KP400652	KP645346	KP735695
*C. verruculosa*	NCK 1675	Human skin	Taiwan	LC802641	LC802651	LC802661
*C. vidyodayana*	USJCC-0029 ^T^	*Oryza sativa*	Sri Lanka	OQ275234	OQ269645	OQ332413
*C. vietnamensis*	FMR 17659 ^T^	unidentified dead leaves	Vietnam	LR736642	LR736644	LR736647
*C. warraberensis*	BRIP 14817 ^T^	*Dactyloctenium aegyptium*	Australia	MH414909	MH433653	MH433672
*C. xishuangbannaensis*	KUMCC 17-0185 ^T^	*Pandanus amaryllifollus*	Thailand	MH275058	MH412750	MH412765
***C. xuanwuensis***	**XW4-33**	**Leaves of *Cynodon dactylon***	**China**	**PV973434**	**PV995194**	**PV995210**
***C. xuanwuensis***	**XW9-10**	**Leaves of *Cynodon dactylon***	**China**	**PV973435**	**PV995195**	**PV995211**
***C. xuanwuensis***	**ZS19-14^T^**	**Leaves of *Cynodon dactylon***	**China**	**PV973436**	**PV995196**	**PV995212**
***C. xuanwuensis***	**ZS19-15**	**Leaves of *Cynodon dactylon***	**China**	**PV973437**	**PV995197**	**PV995213**
*C. yamadana*	COAD 359	*Cyperus rotundus*	Brazil	MN954705	–	MT008260
*Curvularia* sp.	IRAN 4273C	*Saccharum officinarum*	Iran	PP593902	PP661374	PP661357
*Curvularia* sp.	IRAN 3500C	*Saccharum officinarum*	Iran	PP593903	PP661375	PP661358

Phylogenetic trees were inferred using both Maximum Likelihood (ML) and Bayesian Inference (BI) approaches, implemented in RAxML v.8.2.10 (Stamatakis 2014) and MrBayes 3.2.6 (Ronquist et al. 2012), respectively. For the ML analysis, statistical support values were obtained by using rapid bootstrapping with 1000 replicates, with default settings for other parameters. For BI, the optimal partitioning scheme and substitution model were selected with ModelFinder (Kalyaanamoorthy et al. 2017) via its “greedy” algorithm. Branch lengths were linked across partitions and selected by AICc. Four Markov Chain Monte Carlo chains (one cold) were constructed for 5,000,000 generations, with sampling every 1000 generations. Following the burn-in phase (first 25% of sampled trees), the discarded trees were excluded and posterior probabilities (BPP) in the majority rule consensus tree were calculated from the remaining trees.

Phylogenetic trees were visualised by using FigTree version 1.4.4 (Rambaut 2018). Branches that received bootstrap supports for ML (≥ 50%) and BPP (≥ 0.90) were considered as significantly supported.

## ﻿Results

### ﻿Symptoms in the field

**Figure 1.**
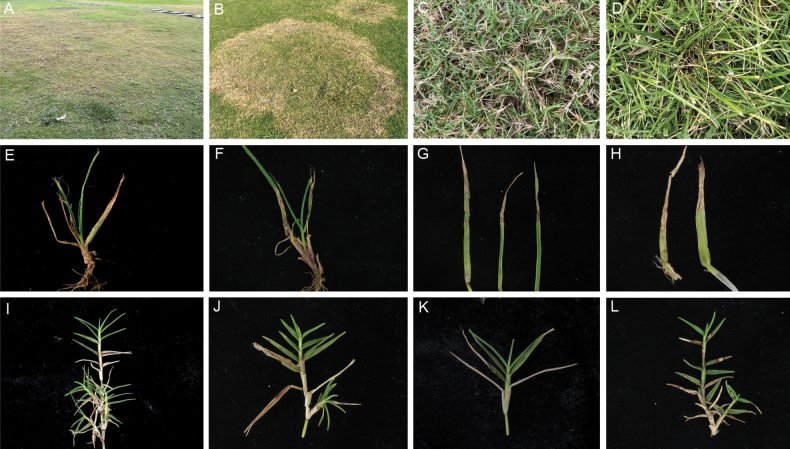
Disease symptoms associated with turfgrass samples (*Cynodon dactylon* and *Lolium perenne*) in this study. **A–D.** Symptoms documented at the site of collection; **E–H.** Detailed view in the collected *Lolium perenne* leaves; **I–L.** Detailed view in the collected *Cynodon dactylon* leaves.

The symptoms of disease caused by *Curvularia* species visible in the field are shown in Fig. 1. On diseased plants, the leaves exhibit progressive yellowing and become densely speckled with yellowish-brown to black necrotic lesions. As the disease advances, these spots coalesce into larger confluent patches, ultimately causing complete leaf wilting and chlorosis. Under humid conditions, the lesion surfaces develop distinct black mould-like structures, composed of fungal mycelia and conidia. These symptoms closely resemble those induced by *Curvularia* strains on turfgrass species, as previously documented in pathological studies (Tomaso-Peterson et al. 2016).

### ﻿Phylogeny

**Figure 2.**
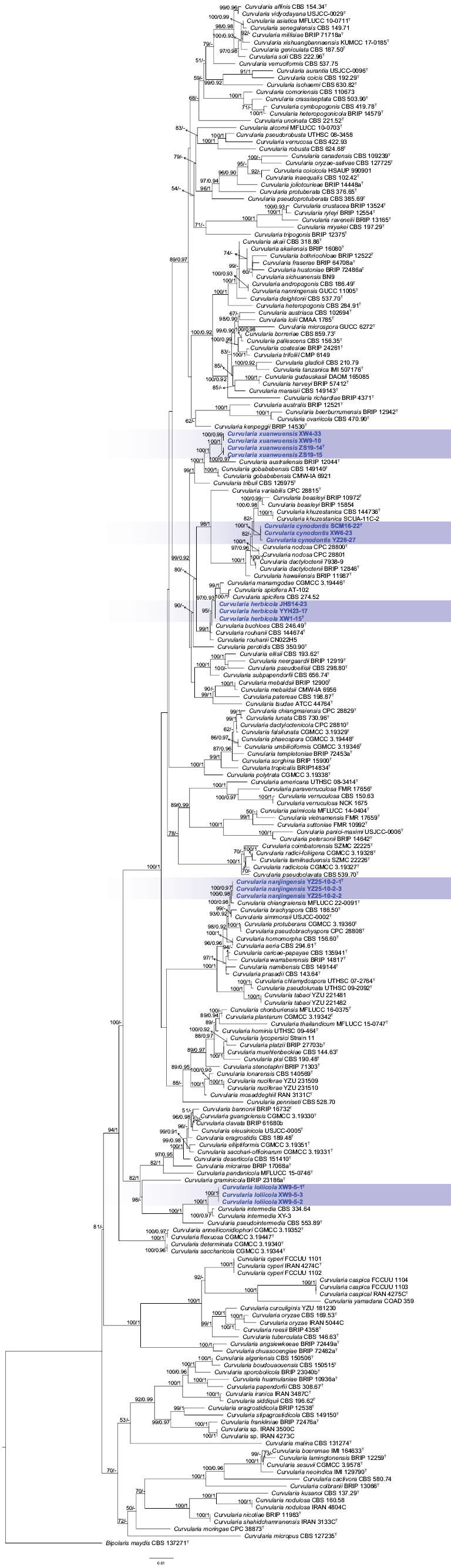
Phylogenetic relationships of *Curvularia* strains isolated from turfgrasses and closely-related taxa of *Curvularia*, based on Maximum Likelihood analyses using concatenated ITS + GAPDH + *tef1* sequences. Numbers above the branches indicate ML bootstrap values (left, ML ≥ 50%) and Bayesian posterior probabilities (right, BPP ≥ 0.90). T = Ex-type strain.

In this study, the concatenated (ITS + GAPDH + *tef1*) dataset included sequences from 217 strains, representing 188 species of *Curvularia*. Sequences of *Bipolaris maydis* (Y. Nisik. & C. Miyake) Shoemaker and used as an outgroup followed Ahmadpour et al. (2025). The final alignment consisted of 2,006 characters (ITS: 471; GAPDH: 552; *tef1*: 983), including gaps. For Bayesian Inference (BI) analysis, the most appropriate models for each locus were confirmed using ModelFinder (The selected models were SYM + I + G4 for ITS, GTR + F + I + G4 for GAPDH and GTR + F + I + G4 for *tef1*). Bayesian analysis resulted in a nearly congruent topology with an average standard deviation of split frequencies as 0.011855 to ML analysis and, thus, only the ML tree is provided (Fig. 2). ML bootstrap support values (BS) ≥ 50% and Bayesian posterior probabilities (BPP) ≥ 0.90 are indicated on the branches.

Phylogenetic analyses revealed five new distinct lineages of *Curvularia* amongst the examined isolates (Fig. 2), ultimately leading to the recognition of five new species, based on morphological characteristics and multi-locus phylogeny (ITS, GAPDH and *tef1*). Sequences of three strains (SCM16-22, XW6-23, YZ26-27) formed a strongly supported lineage, namely *C. cynodontis*, related to *C. beasleyi* Y.P. Tan & R.G. Shivas, *C. khuzestanica* M. Mehrabi, Khodadadi & Farokhinejad and *C. nodosa* Y. Marín, Cheew. & Crous. Sequences of three strains (JHS14-23, YYH23-17, XW1-15) formed an independent lineage, representing novel species *C. herbicola*, sister to the *C. spicifera* (Bainier) Boedijn and *C. manamgodae* Raza, K.D. Hyde & L. Cai. Three strains (YZ25-10-2-1, YZ25-10-2-2, YZ25-10-2-3) formed a robustly supported lineage, that is sister to *C. chiangraiensis* Yasanthika & de Farias and *C. simmonsii* Ferdinandez, Manamgoda & Udayanga, representing a new species *C. nanjingensis*. In addition, three strains (XW9-5-1, XW9-5-2, XW9-5-3) were named *C. loliicola*, sister to *C. intermedia* Boedijn. Sequences of four strains (XW4-33, XW9-10, ZS19-14, ZS19-15), as novel species *C. xuanwuensis*, formed a lineage sister to *C. australiensis* (Bugnic. ex M.B. Ellis) Manamgoda, L. Cai & K.D. Hyde.

### ﻿Taxonomy

#### ﻿ Curvularia cynodontis

Taxon classificationFungiPleosporalesPleosporaceae

Lin Huang, Jia-Mei Zhao & D.W. Li sp. nov.

DAB4779F-C439-55BA-B662-BF33F647FC9C

860700

Fig. 3

##### Holotype.

**China** • Jiangsu Province, Nanjing, Xuanwu District, Shinsemon Park, 32°09'12"N, 118°78'31"E, isolated from leaf blight of *Cynodon dactylon*, 22 June 2024. Holotype: CFCC 72724 is a living specimen being maintained via lyophilisation at the China Forestry Culture Collection Center (CFCC). Ex-type (SCM16-22) is maintained at the Forest Pathology Laboratory, Nanjing Forestry University.

**Figure 3.**
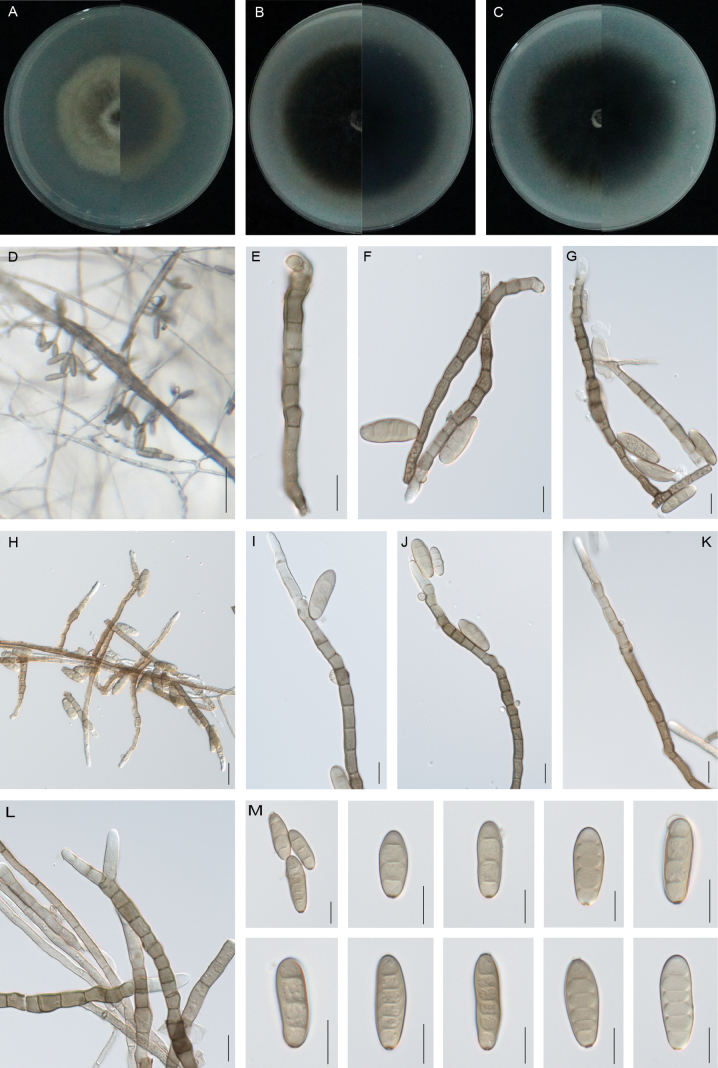
*Curvularia cynodontis* (SCM16-22, type). **A–C.** Colony on PDA (A), OA (B) and CMA (C), respectively in 5 days after incubation at 25 °C; **D.** Sporulation on PDA; **E–J.** Conidia, conidiogenous cells and conidiophores; **K–L.** Conidiophore; **M.** Conidia. Scale bars: 50 μm (**D**); 20 μm (**H**); 10 μm (**E–G**, **I–M**).

##### Etymology.

The name refers to the name of the host genus, *Cynodon*, from which the holotype was collected.

##### Description.

Asexual morph on PDA: **Hyphae** 2–5 μm wide, subhyaline to pale brown, thin- and smooth-walled, septate, branched. **Conidiophores** mononematous, semi- macronematous, septate, arising singly or frequently in groups, straight to slightly flexuous, mostly unbranched, rarely short-branched apically, smooth to verruculose, yellowish-brown to brown, (25–)45–121(–182) × 3–7 μm (mean ± SD = 83 ± 38 × 5 ± 1 μm). **Conidiogenous cells** mono- to polytretic, proliferating sympodially, integrated, terminal or intercalary, subcylindrical to irregularly swollen, brown to dark brown, with darkened scars, smooth, (5–)7–11(–15) × 3–5 μm (mean ± SD = 9 ± 2 × 4 ± 1 μm). **Conidia** 2–5-euseptate, all septa are strongly thickened and swollen, straight, elliptical, smooth, (14–)19–27(–32) × (5–)6–8 μm (mean ± SD = 23 ± 4 × 7 ± 1 μm); germination mono- or bipolar. **Hila** 2–4 μm wide, conspicuous protuberant, thickened and darkened. Chlamydospores and microconidiation not observed. Sexual morph: Undetermined. **Culture characteristics.** Colonies on PDA reach 39 mm diam. after 5 days at 25 °C in the dark. Round colony with regular margins, cottony appearance, fluffy aerial hyphae, grey in the central sporulating zone, white in the marginal aging zone, black in the centre of the reverse and white at the edges. On OA medium, the colonies’ diameter is up to 49 mm, the surface is smooth, the edge is neat and the front and reverse of the colony are black. The back is greyish-black in the centre and white on the edges. On CMA medium, the diameter is up to 49 mm and the colony morphology is similar to that on OA.

##### Additional materials examined.

(All isolated from leaf blight of *Cynodon dactylon*). **China** • Jiangsu Province, Nanjing, Xuanwu District, Xuanwu Lake Park, 21 May 2024, Jia-Mei Zhao and Lin Huang, XW6-23; Jianye District, Yuzui Wetland Park, 29 Aug 2024, Jia-Mei Zhao and Lin Huang, YZ26-27.

##### Notes.

Phylogenetically, *Curvularia cynodontis* forms a distinct, strongly supported lineage, placed in the same clade as *C. beasleyi*, *C. nodosa* and *C. khuzestanica* (Fig. 2). However, these species are readily distinguishable morphologically. *Curvularia beasleyi*, *C. nodosa* and *C. khuzestanica* show uniform width in basal and middle sections, but widen apically, resembling hyphae, whereas *C. cynodontis* maintains consistent conidiophores width distinct from hyphae (Marin-Felix et al. 2017; Tan et al. 2018). Additionally, *C. nodosa* exhibits inconspicuous septate conidia, whereas *C. cynodontis* shows distinctly visible septa in all cells (Marin-Felix et al. 2017). *Curvularia nodosa* and *C. beasleyi* exhibit longer conidiophores than *C. cynodontis* (70–230 μm vs. 45–121 μm in the former, up to 110 μm vs. 45–121 μm in the latter, Marin-Felix et al. (2017); Tan et al. (2018)). In addition, *C. khuzestanica* produces significantly shorter conidiophores than those of *C. cynodontis* (24–90 μm vs. 45–121 μm; Tan et al. (2018)).

#### ﻿ Curvularia herbicola

Taxon classificationFungiPleosporalesPleosporaceae

Lin Huang, Jia-Mei Zhao & D.W. Li sp. nov.

5F8031E7-0897-50C0-8841-B8B158F93DD8

860701

Fig. 4

##### Holotype.

**China** • Jiangsu Province, Nanjing, Jianye District, Xuanwu District, Xuanwu Lake Park, 32°06'79"N, 118°80'83"E, isolated from leaf blight of *Cynodon dactylon*, 21 May 2024. Holotype: CFCC 72734 is a living specimen being maintained via lyophilisation at the China Forestry Culture Collection Center (CFCC). Ex-type (XW1-15) is maintained at the Forest Pathology Laboratory, Nanjing Forestry University.

**Figure 4.**
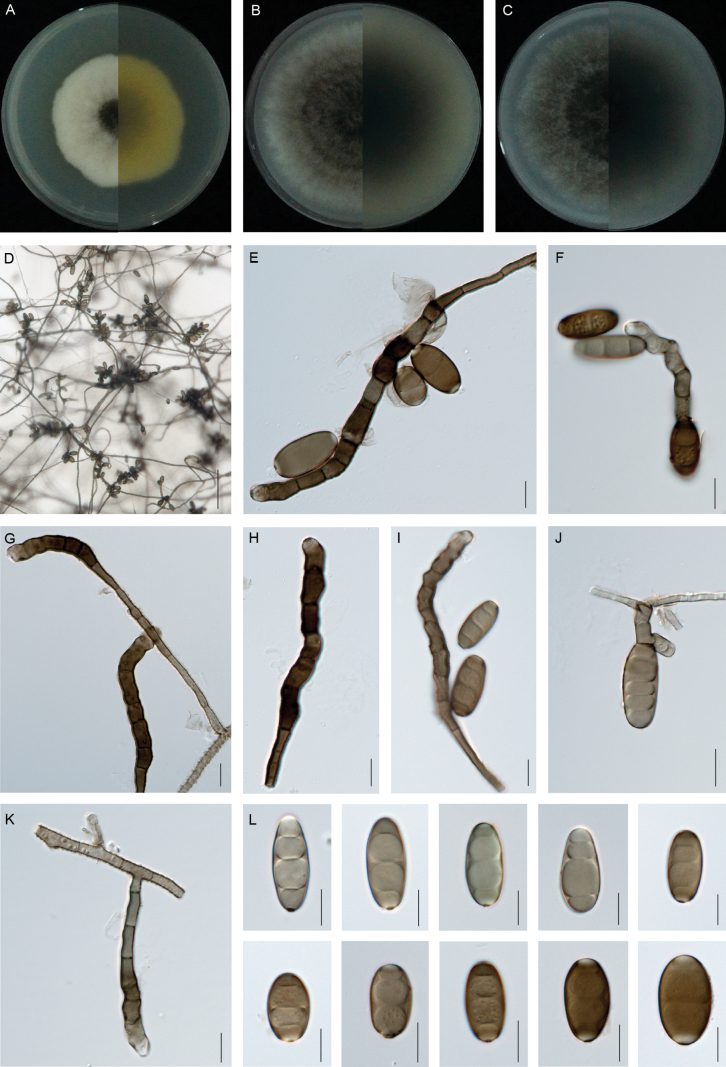
*Curvularia herbicola* (XW1-15, type). **A–C.** Colony on PDA (**A**), OA (**B**) and CMA (**C**), respectively in 5 days after incubation at 25 °C; **D.** Sporulation on PDA; **E–F, I–J.** Conidia, conidiogenous cells and conidiophores; **G–H, K.** Conidiogenous cell and conidiophore; **L.** Conidia. Scale bars: 100 μm (**D**); 10 μm (**E–L**).

##### Etymology.

The name, Latin nouns for “grass”, herba + Latin term -cola (inhabitant or dweller) refers to grass, which this species inhabits.

##### Description.

Asexual morph on PDA: **Hyphae** 2–4 μm wide, subhyaline to pale brown, verruculose to verrucose, septate, branched. **Conidiophores** mononematous, semi-to macronematous, septate, arising singly or frequently in groups, straight to flexuous, geniculate towards the apex, unbranched, smooth to verruculose, yellowish-brown to brown, (50–)66–136 × 2–4 μm (mean ± SD = 101 ± 35 × 3 ± 1 μm). **Conidiogenous cells** mono- to polytretic, proliferating sympodially, integrated, terminal or intercalary, conspicuously swollen, brown to dark brown, pale brown initially, becoming distinctly darker, with darkened scars, smooth, (5–)7–11(–14) × 5–7(–10) μm (mean ± SD = 9 ± 2 × 6 ± 1 μm). **Conidia** straight, elliptical, smooth, 2–4-euseptate, with strongly thickened and swollen, septa (16–)19–25(–29) × (8–)9–11 μm (mean ± SD = 22 ± 3 × 10 ± 1 μm); germination mono- or bipolar. **Hila** 2–3 μm wide, conspicuous protuberant, thickened and darkened. Chlamydospores and microconidiation not observed. Sexual morph: Undetermined.

##### Culture characteristics.

Colonies on PDA reach 39 mm diam. after 5 days at 25 °C in the dark. Round colony with slightly irregular margins, the central sporulating zone is black and flat, the margins are aged and raised, cottony appearance, yellowish-black at the sporulating zone and yellowish-white at the aging zone. On OA medium, the colonies reach 60 mm in diam., which is cotton wool-like, with neat edges, grey-black surface, grey-white edges and fluffy aerial hyphae. The back is greyish-white in the centre and white on the edges. On CMA medium, the diameter is up to 57 mm and the colony morphology is similar to that on OA.

##### Additional materials examined.

**China** • Jiangsu Province, Nanjing, Xuanwu District, Jiuhuashan Park, 18 June 2024, JHS14-23; Qinhuai District, Yueyahu Park, YYH23-17, isolated from leaf blight of *Lolium perenne* and *Cynodon dactylon*, respectively, Jia-Mei Zhao and Lin Huang.

##### Notes.

*Curvularia herbicola* is phylogenetically sister to *C. manamgodae* and *C. spicifera* (Fig. 2). Morphologically, *C. manamgodae* can be easily distinguished from *C. herbicola* by its larger conidiophores (38–550 × 4–8 μm vs. 66–136 × 2–4 μm; Laforet (2015)). *Curvularia spicifera* differs from *C. herbicola* by its longer conidia (20–40 μm vs. 19–25 μm, Raza et al. (2019)). Furthermore, *C. manamgodae* was isolated from the leaf lesions of sugarcane. *C. spicifera* was isolated from some crops such as rice, corn and sorghum and reported to cause leaf spot disease and wilt disease (Krizsan et al. 2015; Raza et al. 2019; Aslam et al. 2020; Ram et al. 2024).

#### ﻿ Curvularia loliicola

Taxon classificationFungiPleosporalesPleosporaceae

Lin Huang, Jia-Mei Zhao & D.W. Li sp. nov.

EF95DB15-DAFD-539A-B8A5-8897D7C0AD3B

860702

Fig. 5

##### Holotype.

**China** • Jiangsu Province, Nanjing, Xuanwu District, Xuanwu Lake Park, 32°06'62"N, 118°80'54"E, isolated from leaf blight of *Lolium perenne*, 5 June 2024. Holotype: CFCC 72725 is a living specimen being maintained via lyophilisation at the China Forestry Culture Collection Center (CFCC). Ex-type (XW9-5-1) is maintained at the Forest Pathology Laboratory, Nanjing Forestry University.

**Figure 5.**
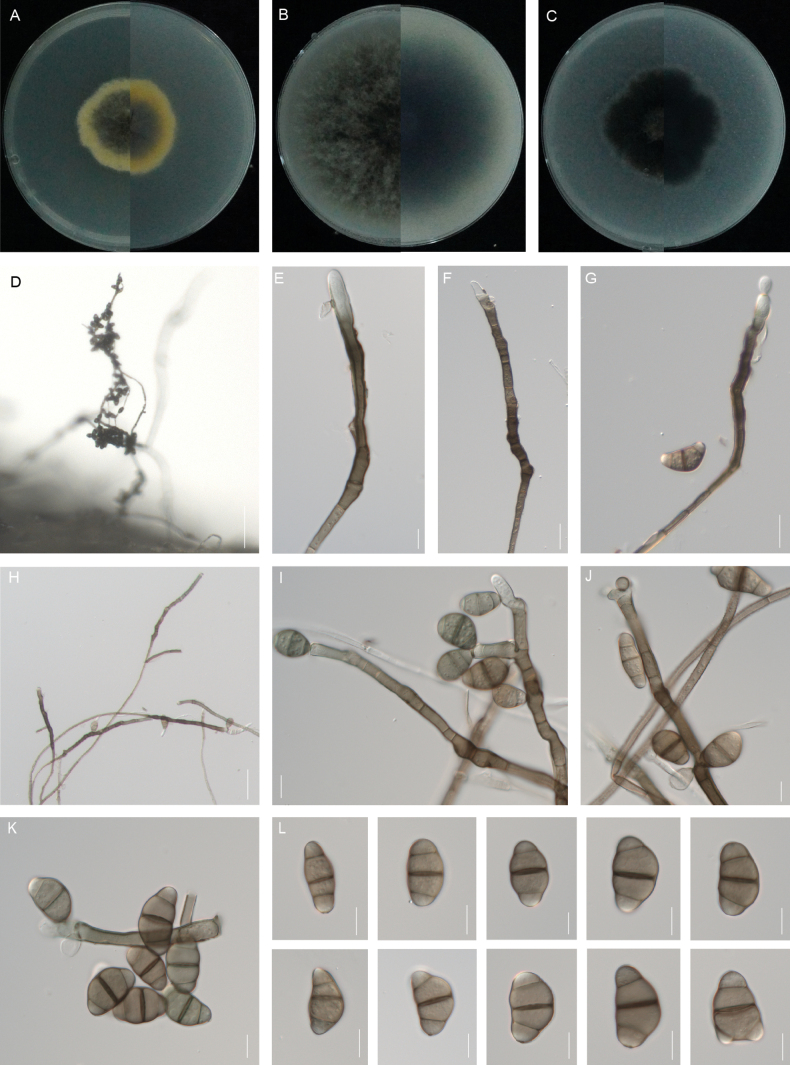
*Curvularia loliicola* (XW9-5-1, type). **A–C.** Colony on PDA (**A**), OA (**B**) and CMA (**C**), respectively in 5 days after incubation at 25 °C; **D.** Sporulation on PDA; **E–F.** Conidiogenous cell and conidiophore; **G–K.** Conidia, conidiogenous cell and conidiophore; **L.** Conidia. Scale bars: 100 μm (**D**); 50 μm (**H**); 10 μm (**E–G**, **I–L**).

##### Etymology.

The name refers to the genus of the host, *Lolium*, from which the holotype was collected.

##### Description.

Asexual morph on PDA: **Hyphae** 3–5 μm wide, subhyaline to pale brown, thin and verruculose, septate, branched. **Conidiophores** mononematous, semi- to macronematous, septate, arising singly or frequently in groups, straight to flexuous, geniculate at upper part septate, mostly unbranched, rarely short-branched apically, smooth, pale brown to brown, paler towards the apex, (75–)97–155(–192) × (4–)6–8(–11) μm (mean ± SD = 126 ± 29 × 7 ± 1 μm). **Conidiogenous cells** mono- to polytretic, proliferating sympodially, integrated, terminal or intercalary, subcylindrical to slightly swollen, yellowish-brown, smooth to slightly verruculose, with thickened and darkened scars, (8–)11–25(–30) × (3–)5–7(–8) μm (mean ± SD = 18 ± 7 × 6 ± 1 μm). **Conidia** straight or curved, pale brown to dark golden brown, smooth, fusiform, 2–3-euseptate, median two cells asymmetrically swollen, median septum thickened, 23–29(–32) × (11–)12–16 μm (mean ± SD = 26 ± 3 × 14 ± 2 μm); germination mono- or bipolar. **Hila** 2–3 μm wide, non-protuberant, thickened and darkened. Chlamydospores and microconidia are not observed. Sexual morph not observed.

##### Culture characteristics.

Colonies on PDA reach 33 mm diam. after 5 days at 25 °C in the dark. Margins irregular, umbonate with an erose edge, greyish-green, cottony appearance, yellowish on the periphery, flat. The centre of the reverse is greyish-green and the edge is dark yellow. On OA medium, the colonies reach up to 47 mm in diam. The edge is neat, cottony appearance, the colony surface is grey, aerial hyphae are fluffy and reverse is black. On CMA medium, the colonies are cottony and reach 32 mm in diam. The edge is irregular and the centre is black.

##### Additional materials examined.

**China** • Jiangsu Province, Nanjing, Xuanwu District, Xuanwu Lake Park, isolated from leaf blight of *Lolium perenne*, 5 June 2024, Jia-Mei Zhao and Lin Huang, XW9-5-2, XW9-5-3.

##### Notes.

Phylogenetically, *Curvularia loliicola* forms a distinct, strongly supported lineage sister to *C. intermedia* (Fig. 2). Morphologically, *C. loliicola* resembles *C. intermedia* in producing 3-septate conidia, but differs in conidial and conidiophore size and ornamentation. *Curvularia intermedia* possesses longer conidia (33–37 vs. 23–29 μm in length). In addition, *C. intermedia* distinctly differs from *C. loliicola* by its longer conidiophores which can reach up to 800 μm, while the conidiophores of *C. loliicola* show no basal enlargement and are semi- to macro-nematous, up to 192 μm (Ahmadpour et al. 2012). Furthermore, it has also been reported that it can cause leaf spots of *Cynodon dactylon*, blueberries and other plants (Couch 1995; Li et al. 2019; Cheng et al. 2022; Kong et al. 2024). In addition, the novel species resembles *Curvularia graminis* Meng Zhang & T.Y. Zhang, described from grass hosts in China, particularly in conidial morphology, where the second and third septa from the base are often darker pigmented. However, *C. graminis* differs from *C. loliicola* in having longer conidia (29–46 vs. 23–29 μm, Zhang and Zhang (2007)).

#### ﻿ Curvularia nanjingensis

Taxon classificationFungiPleosporalesPleosporaceae

Lin Huang, Jia-Mei Zhao & D.W. Li sp. nov.

32890A52-C0D0-52BD-B74E-55FCCE196262

860703

Fig. 6

##### Holotype.

**China** • Jiangsu Province, Nanjing, Jianye District, Yuzui Wetland Park, 31°97'13"N, 118°65'54"E, isolated from leaf blight of *Cynodon dactylon*, 29 Aug 2024. Holotype: CFCC 72723 is a living specimen being maintained via lyophilisation at the China Forestry Culture Collection Center (CFCC). Ex-type (YZ25-10-2-1) is maintained at the Forest Pathology Laboratory, Nanjing Forestry University.

**Figure 6.**
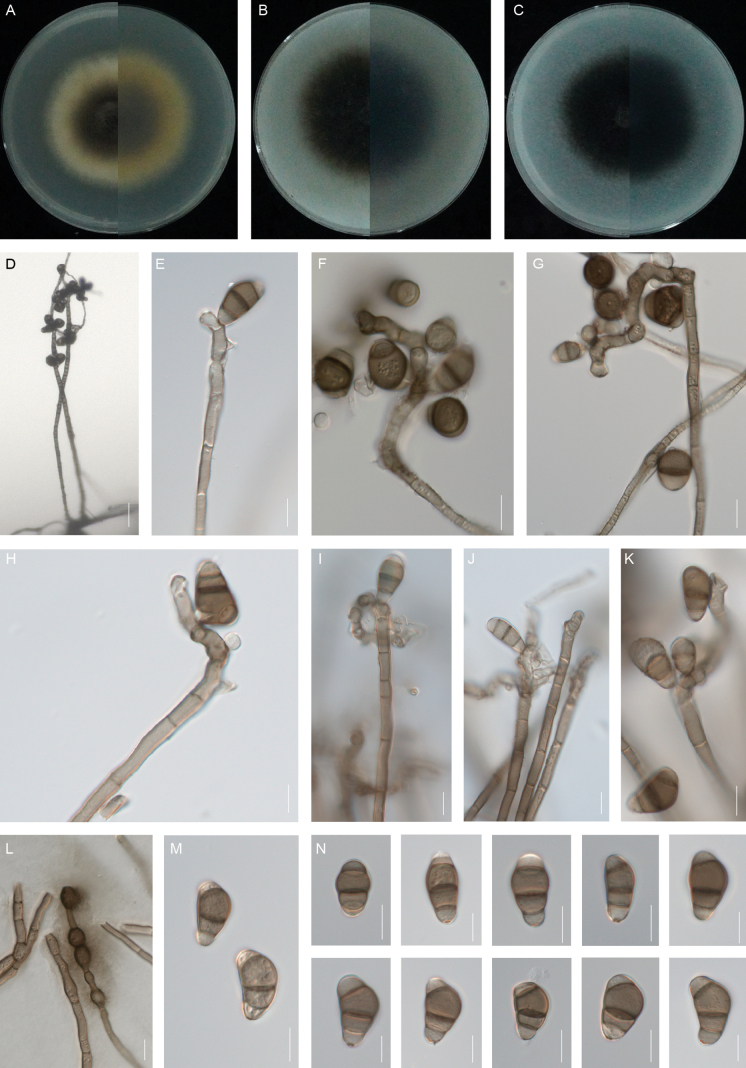
*Curvularia nanjingensis* (YZ25-10-2-1, type). **A–C.** Colony on PDA (**A**), OA (**B**) and CMA (**C**), respectively in 5 days after incubation at 25 °C; **D.** Sporulation on PDA; **E–K.** Conidia, conidiogenous cells and conidiophore; **L.** Chlamydospores; **M–N.** conidia. Scale bars: 100 μm (**D**); 10 μm (**H–M**).

##### Etymology.

The name refers to the city, Nanjing where the holotype was collected.

##### Description.

Asexual morph on PDA: **Hyphae** 3–4 μm wide, subhyaline to pale brown, thin- and smooth-walled, septate, branched. **Conidiophores** mononematous, semi- to macronematous, septate, arising singly or frequently in groups, straight to flexuous, geniculate towards the apex, unbranched, smooth-walled, subhyaline to dark brown, (113–)163–279(–384) × (3–)4–6 μm (mean ± SD = 221 ± 58 × 5 ± 1 μm). **Conidiogenous cells** mono- to polytretic, proliferating sympodially, integrated, terminal or intercalary, subcylindrical to irregularly swollen, yellowish-brown, smooth, 6–14(–18) × 4–6 μm (mean ± SD = 10 ± 4 × 5 ± 1 μm). **Conidia** straight or curved, elliptical to lunate, smooth, 2–3-euseptate, median cells unequally enlarged, pigmentation intensified to dark brown and median septum thickened, apical and basal cells subhyaline to pale brown, (13–)16–20(–22) × (7–)9–13(–14) μm (mean ± SD = 18 ± 2 × 11 ± 2 μm); germination mono- or bipolar. **Hila** 2–3 μm wide, inconspicuous to slightly conspicuous, slightly thickened and darkened. **Chlamydospores** present, intercalary, smooth-walled, solitary or grouped in chain, subglobose to oblong, 8–12(–13) × (6–)7–9(–10) μm (mean ± SD = 10 ± 2 × 8 ± 1 μm, n = 20). Microconidia not observed. Sexual morph: Undetermined.

##### Culture characteristics.

Colonies on PDA reach 46 mm diam. after 5 days at 25 °C in the dark. Round colonies, regular margin, flat without bulge, greyish-black at sporulating zone, white at aging zone. The centre of the reverse is greyish-green and the edge is yellowish-white. On OA medium, the colonies are up to 51 mm in diam., with regular margin, smooth surface, black on both front and reverse sides. On CMA medium, the diameter is up to 49 mm and the morphology is similar to that on OA medium.

##### Additional materials examined.

**China** • Jiangsu Province, Nanjing, Jianye District, Yuzui Wetland Park, 29 August 2024, isolated from leaf blight of *Cynodon dactylon*, Jia-Mei Zhao and Lin Huang, YZ25-10-2-2, YZ25-10-2-3.

##### Notes.

Phylogenetically, *Curvularia nanjingensis* is allocated to a strongly supported lineage (100/0.98) in *Curvularia* and related to *C. chiangraiensis* and *C. simmonsii* (Fig. 2). However, *C. chiangraiensis* can be easily distinguished from *C. nanjingensis* by its smaller conidiophores (50–150 × 2–7 μm vs. 163–279 × 4–6 μm, Yasanthika et al. (2023)). *Curvularia simmonsii* differs from *C. nanjingensis* by its longer conidia (21–27 μm vs. 16–20 μm, Ferdinandez et al. (2021)). Additionally, *C. nanjingensis* can produce chlamydospores, while this feature is not observed in *C. chiangraiensis* and *C. simmonsii*. Furthermore, *C. chiangraiensis* and *C. simmonsii* have only been sporadically reported. The former has been reported to be isolated from soil; the latter was isolated from the leaf lesions of *Panicum maximum* in Sri Lanka (Ferdinandez et al. 2021; Yasanthika et al. 2023).

#### ﻿ Curvularia xuanwuensis

Taxon classificationFungiPleosporalesPleosporaceae

Lin Huang, Jia-Mei Zhao & D.W. Li sp. nov.

4C4B4237-271E-5763-A4C0-D6C5AF88A3E6

860704

Fig. 7

##### Holotype.

**China** • Jiangsu Province, Nanjing, Xuanwu District, Zhongshan Sports Park, 32°05'03"N, 118°86'69"E, isolated from leaf blight of *Cynodon dactylon*, 3 July 2024. Holotype: CFCC 72729 is a living specimen being maintained via lyophilisation at the China Forestry Culture Collection Center (CFCC). Ex-type (ZS19-14) is maintained at the Forest Pathology Laboratory, Nanjing Forestry University.

**Figure 7.**
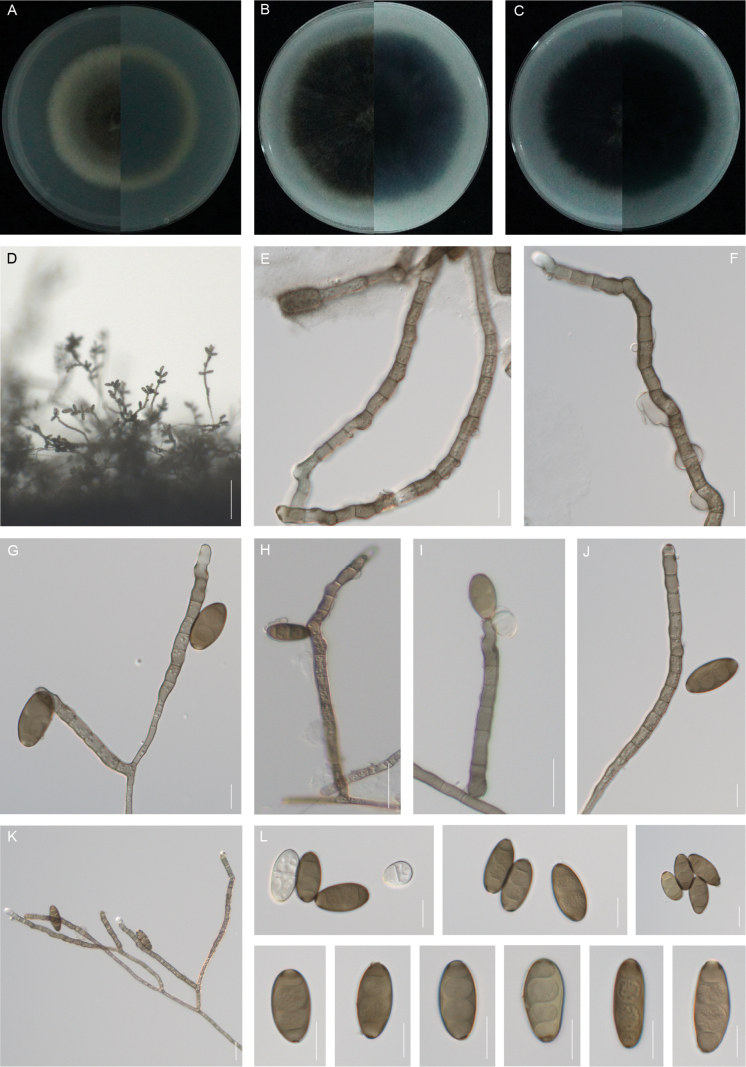
*Curvularia xuanwuensis* (ZS19-14, type). **A–C.** Colony on PDA (**A**), OA (**B**) and CMA (**C**), respectively in 5 days after incubation at 25 °C; **D.** Sporulation on PDA; **E.** Conidiogenous cell and conidiophore; **F–K.** Conidia, conidiogenous cells and conidiophores; **L.** Conidia. Scale bars: 100 μm (**D**); 20 μm (**H–I, K**); 10 μm (**E–G, J, L**).

##### Etymology.

The epithet name after the Xuanwu District where the holotype of the fungus was collected.

##### Description.

Asexual morph on PDA: **Hyphae** 2–4 μm wide, subhyaline to pale brown, thin- and smooth-walled hyphae, septate, branched. **Conidiophores** mononematous, semi- to macronematous, septate, arising singly or frequently in groups, straight to flexuous, geniculate at upper part, unbranched, smooth, pale brown to brown, paler towards the apex, (49–)60–180(–239) × (3–)4–6(–7) μm (mean ± SD = 120 ± 60 × 5 ± 1 μm). **Conidiogenous cells** mono- to polytretic, proliferating sympodially, integrated, terminal or intercalary, subcylindrical, yellowish-brown, smooth to slightly verruculose, with thickened and darkened scars, (6–)7–11(–14) × 4–6(–7) μm (mean ± SD = 9 ± 2 × 5 ± 1 μm). **Conidia** straight, pale brown to dark golden brown, smooth, ellipsoidal, 2–3-euseptate, (14–)18–22(–24) × (7–)8–10 μm (mean ± SD = 20 ± 2 × 9 ± 1 μm); germination mono- or bipolar. **Hila** 2–3 μm wide, non-protuberant, thickened and darkened. Chlamydospores and microconidia were not observed. Sexual morph not observed.

##### Culture characteristics.

Colonies on PDA reach 44 mm diameter after 5 days at 25 °C in the dark. Round colonies with an entire margin, greyish-black at sporulating zone, flat and white at aging zone, cottony appearance. The centre of the reverse is greyish-green and the edge is white. On OA medium, the colonies attain up to 51 mm in diam. The edge is neat, cottony appearance, the surface of the colony is grey-black, the edge is white and the aerial hyphae are fluffy. Reverse side is greyish-black in the centre and white on the edges. On CMA medium, the colony is up to 49 mm in diam., with an irregular margin, the front and back sides are black and the surface is smooth.

##### Additional materials examined.

**China** • Jiangsu Province, Nanjing, Xuanwu District, Zhongshan Sports Park, 3 July 2024, isolated from leaf blight of *Cynodon dactylon*, Jia-Mei Zhao and Lin Huang, ZS19-15; Xuanwu Lake Park, 5 June 2024, isolated from leaf blight of *Cynodon dactylon*, Jia-Mei Zhao and Lin Huang, XW4-33, XW9-10.

##### Notes.

Our phylogenetic analyses reveal that *Curvularia xuanwuensis* forms a strongly supported (100/1) lineage and sisters to *C. australiensis*. However, these species are readily distinguishable in morphology. *C. australiensis* differs from *C. xuanwuensis* by its longer conidia (15–40 μm vs. 18–22 μm), with thicker and more conspicuous conidial septa and larger conidiophores (95–205 × 3–7 μm vs. 60–180 × 4–6 μm, Laforet (2015)).

## ﻿Discussion

Species identification within the genus *Curvularia* traditionally relies on morphological characteristics, encompassing shape and length of conidiophores and conidia, as well as the presence of structures like hilum, stroma, chlamydospores and microconidia (Tan et al. 2014, 2018; Manamgoda et al. 2015; Marin-Felix et al. 2020). However, reliable species distinction is challenging due to many overlapping morphological characters (Sivanesan 1987; Alcorn 1988; Manamgoda et al. 2012, 2015). Studies have reported multiple isolation of *Curvularia* species from a single plant in different geographic locations, further confirming the complexity of *Curvularia* species delineation (Ahmadpour et al. 2014; Heidarian et al. 2020; Ferdinandez et al. 2021, 2023). Therefore, phylogenetic inference, based on DNA sequence data, is essential. Furthermore, ITS sequences alone are insufficient to accurately differentiate individual species and phylogenetic analyses using multi-locus ITS + GAPDH + *tef1* genomic loci provide the highest resolution for species boundaries amongst *Curvularia* species (Berbee et al. 1999; Kiss et al. 2020; van Vuuren et al. 2024; Ahmadpour et al. 2025; Guo et al. 2025). Consequently, the modern taxonomic system for *Curvularia* is based on morphology and multi-locus phylogenetic analyses. In this study, five new species of *Curvularia* are identified in Jiangsu Province, China: *C. cynodontis*, *C. herbicola*, *C. loliicola*, *C. nanjingensis* and *C. xuanwuensis*.

*Curvularia* species exhibit a wide range of ecological roles, including plant, animal or human pathogens, as well as epiphytes, saprobes or endophytes, predominantly associated with cultivated cereals (Manamgoda et al. 2015; Marin-Felix et al. 2020; Farr et al. 2021; Ahmadpour et al. 2025). Amongst them, as the phytopathogens, *Curvularia* species were primarily associated with grasses of the Poaceae, such as major grains like rice, wheat and corn, as well as ornamental plants like turfgrasses (Smiley et al. 2005; Zhang et al. 2020b; Tredway et al. 2023; Ram et al. 2024). In this study, based on multi-locus phylogenetic analyses and morphological attributes, five new *Curvularia* species were described from ornamental Poaceae plants in China. *Curvularia cynodontis*, *C. xuanwuensis* and *C. nanjingensis* were only isolated from *Cynodon dactylon* and *C. cynodontis* was the most frequently isolated species in this study, including 11 strains. In addition, of other species described in this study, *C. loliicola* was found only in the leaves of *Lolium perenne*, while *C. herbicola* was isolated from these two grass plants.

*Curvularia* is a cosmopolitan genus, with more than 30 first reports on different plants in China, India and Pakistan found in literature from 2010 to the present (Zhang et al. 2020b). It is evident that *Curvularia* species have maintained a close association with plant diversity. Although this study revealed associations between specific *Curvularia* species and particular substrates (*Cynodon dactylon* and *Lolium perenne*), broader sampling would help better understood their biology, substrate preferences, and distribution patterns.

Collectively, this study contributes to a better understanding of the diversity of *Curvularia* isolates associated with Poaceae hosts. Further, this study provides valuable insights for plant pathologists, mycologists, agronomists and environmental scientists with actionable insights to enhance conservation and management strategies for key Poaceae ornamentals or crops.

## Supplementary Material

XML Treatment for Curvularia cynodontis

XML Treatment for Curvularia herbicola

XML Treatment for Curvularia loliicola

XML Treatment for Curvularia nanjingensis

XML Treatment for Curvularia xuanwuensis

## ﻿Additional information

### Use of AI

No use of AI was reported.

### Author ORCIDs

Jia-Mei Zhao https://orcid.org/0009-0005-6748-8682

Qiu-Yue Zhang https://orcid.org/0000-0001-9458-3566

De-Wei Li https://orcid.org/0000-0002-2788-7938

Lin Huang https://orcid.org/0000-0001-7536-0914

### Data availability

All of the data that support the findings of this study are available in the main text or Supplementary Information.

## Supplementary materials

Supplementary material 1 Supplementary tables S1, S2 Data type xlsx https://binary.pensoft.net/file/1472461This dataset is made available under the Open Database License (http://opendatacommons.org/licenses/odbl/1.0/). The Open Database License (ODbL) is a license agreement intended to allow users to freely share, modify, and use this Dataset while maintaining this same freedom for others, provided that the original source and author(s) are credited.Jia-Mei Zhao, Chuan-Xu Peng, Qiu-Yue Zhang, De-Wei Li, Lin Huang
